# Differential Effects of Dry vs. Wet Heating of β-Lactoglobulin on Formation of sRAGE Binding Ligands and sIgE Epitope Recognition

**DOI:** 10.3390/nu11061432

**Published:** 2019-06-25

**Authors:** Hannah E. Zenker, Arifa Ewaz, Ying Deng, Huub F. J. Savelkoul, R.J. Joost van Neerven, Nicolette W. De Jong, Harry J. Wichers, Kasper A. Hettinga, Malgorzata Teodorowicz

**Affiliations:** 1 Food Quality & Design Group, Wageningen University & Research Centre, 6700 AA Wageningen, The Netherlands; kasper.hettinga@wur.nl; 2 Cell Biology & Immunology, Wageningen University & Research Centre, 6700 AA Wageningen, The Netherlands; arifa.ewaz@wur.nl (A.E.); huub.savelkoul@wur.nl (H.F.J.S.); joost.vanneerven@frieslandcampina.com (R.J.J.v.N.); gosia.teodorowicz@wur.nl (M.T.); 3 Wageningen Food & Biobased Research, Wageningen University & Research Centre, 6700 AA Wageningen, The Netherlands; ying.deng@wur.nl (Y.D.); harry.wichers@wur.nl (H.J.W.); 4 Laboratory of Food Chemistry, Wageningen University and Research, 6700 AA Wageningen, The Netherlands; 5 FrieslandCampina, 3800 BN Amersfoort, The Netherlands; 6 Erasmus University medical Centre Rotterdam, Dept. Internal Medicine, 3000 CA Rotterdam, The Netherlands; n.w.dejong@erasmusmc.nl

**Keywords:** aggregation, allergenicity, β-lactoglobulin, CML, glycation, sRAGE, IgE binding

## Abstract

The effect of glycation and aggregation of thermally processed β-lactoglobulin (BLG) on binding to sRAGE and specific immunoglobulin E (sIgE) from cow milk allergic (CMA) patients were investigated. BLG was heated under dry conditions (water activity < 0.7) and wet conditions (in phosphate buffer at pH 7.4) at low temperature (<73 °C) and high temperatures (>90 °C) in the presence or absence of the milk sugar lactose. Nε-(carboxymethyl)-l-lysine (CML) western blot and glycation staining were used to directly identify glycation structures on the protein fractions on SDS-PAGE. Western blot was used to specify sRAGE and sIgE binding fractions. sRAGE binding was highest under wet-heated BLG independent of the presence of the milk sugar lactose. Under wet heating, high-molecular-weight aggregates were most potent and did not require the presence of CML to generate sRAGE binding ligands. In the dry system, sRAGE binding was observed only in the presence of lactose. sIgE binding affinity showed large individual differences and revealed four binding profiles. Dependent on the individual, sIgE binding decreased or increased by wet heating independent of the presence of lactose. Dry heating required the presence of lactose to show increased binding to aggregates in most individuals. This study highlights an important role of heating condition-dependent protein aggregation and glycation in changing the immunogenicity and antigenicity of cow’s milk BLG.

## 1. Introduction

The manufacturing of many dairy products implies heating at moderate (<73 °C) or high temperatures (>90 °C) to ensure product safety or to produce powdered products such as infant formula. Maillard reaction (MR)-induced glycation, as well as protein aggregation, are the most abundant protein modifications during milk processing. The formation of aggregates and the degree of glycation strongly depends on the applied processing conditions and may strongly differ between wet and dry heating conditions as they are applied in industrial processing of milk or dried dairy products. Wet heating of β-lactoglobulin (BLG) results in relatively more aggregation and the formation of polymers with a molecular weight (MW) > 10 kDa compared to dry heating [1]. At the same time, dry heating induces more glycation as low moisture content (water activity between 0.2 and 0.8) enhances protein glycation via the MR [2]. These protein modifications are known to change reactivity of food allergens to innate cell surface receptors and specific immunoglobulin E (sIgE) binding affinity by either destruction and masking of epitopes or formation of neo-epitopes via exposure of interior structures and amino acid side chain modifications [3]. The applied processing conditions, such as pH, humidity, and temperature are crucial determinants for epitope destruction/formation. For example, pasteurization of cow’s milk BLG enhances its allergenicity by redirecting its epithelial uptake towards Peyer’s patches [4]. At the same time, clinical studies report that “baked milk products” can be tolerated by ~70% of children with IgE-mediated cow’s milk allergy and that consumption of these products potentially facilitates the development of oral tolerance towards raw milk [5,6,7]. Both glycation and aggregation result in decreased binding of sIgE from cow’s milk allergic (CMA) children if heated above 90 °C in a wet system [8,9]. At the same time, cellular signaling can be promoted by the formation of glycation structures on the food allergen [10].

The receptor for advanced glycation end products (RAGE) is one of the most studied cell surface receptors in relation to protein glycation. It is known to bind to advanced glycation end products (AGEs) that are formed via the MR. RAGE is expressed by vascular endothelial cells and cells of the innate and adaptive immune system [10]. RAGE-ligand interaction activates an intracellular signaling cascade, resulting in the release of pro-inflammatory cytokines via NF-κB activation. Its soluble isoform (sRAGE) binds to the same ligands; however it is considered to be a decoy for RAGE in the peripheral system [11]. Besides AGEs, sRAGE binds to several other ligands, such as lipopolysaccharide, amyloid-β, and S100 protein. Those ligands have the common property to act as oligomers [12]. Liu et al. [13] indicated that not only the level of glycation but also the aggregation occurring during dry heating of BLG can promote sRAGE binding affinity. Perkins et al. [14] recently reported the involvement of RAGE in type 2 cytokine signal transduction in the lungs of mice. Type 2 cytokines, e.g., interleukin (IL)-4 are involved in B-cell class switch and sIgE production [15]. These findings indicate the direct involvement of RAGE with the clinical manifestation of the allergic reaction towards the allergen. MR not only leads to the formation of AGEs, as commonly described RAGE ligands, but also promotes protein aggregation [16]. By using controlled processing conditions, protein modification can be directed towards either the formation of AGEs or the formation of aggregates enabling a distinction between the functional effects of these two distinct protein modifications. This study aimed to investigate the effect of protein aggregation and glycation on sRAGE binding affinity and the potential allergenic impact. Therefore, BLG was heated under controlled wet and dry conditions to investigate whether AGE formation or rather aggregation (glycation or non-glycation-induced) contributes to changes in sRAGE and sIgE binding affinity to BLG.

## 2. Materials and Methods

### 2.1. Chemicals

Acetonitril uHPLC-MS grade was purchased from VWR chemicals (Radnor, PA, USA). Nε-(Carboxymethyl)-l-lysine (CML) and Nε-(Carboxy [2H2]methyl)-l-lysine (d2-CML) were purchased from Polypeptide laboratories (Strasbourg, France) NuPAGE^®^ LDS sample buffer (4× conc.), and NuPAGE™ MOPS SDS running buffer (20×), NuPAGE™ 12% Bis-Tris protein gel, 1.0 mm. gels. Soluble AGE Product-Specific Receptor Human *E. coli* (RD172116100) was obtained from Biovendor (Brno, Czech Republic). Anti-RAGE antibody (monoclonal mouse IgG_2_B clone, MAB11451) purchased from R&D systems (Minneapolis, MN, USA). HRP conjugated anti-mouse polyclonal goat (P0447) was purchased from Dako (Glostrup, Denmark). TMB substrate (3,3′,5,5′-tetramethylbenzidine) for high sensitivity ELISA was purchased from sdt-reagents (Baesweiler, Germany). WesternBright^TM^ ECL western blotting detection kit was obtained from Advansta (San Jose, CA, USA). Ovalbumin (OVA) was purchased from InvivoGen (San Diego, CA, USA). Bovine Serum Albumin Fraction V (BSA) was obtained from Roche (Basel, Switzerland). Amyloid-β (1-42) ultrapure HFIP was purchased from Westburg (Leusden, The Netherlands). (N-Epsilon)-Carboxymethyl-Lysine primary antibody was purchased from Nordic-MUbio (Susteren, The Netherlands). Pro-Q^®^ Emerald 300 Glycoprotein Gel and Blot Stain Kit (Thermo Fisher Scientific, Waltham, MA, USA).

BlueRay prestained protein marker was obtained from Jena Bioscience GmbH (Jena, Germany). Coomassie brilliant blue R-250 was purchased from Bio-Rad (Hercules, CA, USA). Ultrapure water was prepared by an Purelab^®^ Ultra water system from ELGA LabWaters (Celle, Germany). Three Plasma were purchased from PlasmaLab International (Everett, WA, USA). Two sera were provided by the Queen Beatrix Hospital (Winters Wijk, The Netherlands), all other sera were provided by the archival serumbank of the Erasmus Medical Centre (Rotterdam, The Netherlands). All other chemicals were purchased from Sigma Aldrich (St Louise, MO, USA) unless mentioned otherwise.

### 2.2. BLG Isolation and Purification

Raw bulk milk obtained from the Department of Animal Sciences, Wageningen University & Research (Wageningen, The Netherlands). BLG was purified and isolated as described by De Jongh et al. [17] using anion exchange chromatography DEAE Sepharose C-6B (GE healthcare, Chicago, IL, USA). Isolated BLG was lyophilised and a purity >94% was measured as described by Deng et al. [1].

### 2.3. Heat Treatment of BLG

BLG was heated in a wet system above the denaturation temperature by heating it in phosphate buffer (PBS) at pH 7.4 applying 100 °C for 90 min in the presence of lactose (W-HT-La) and in the absence of lactose (W-HT). Three additional treatments were conducted as described by Deng et al. [1]. Briefly, a low-temperature wet heating was conducted by heating BLG below its denaturation temperature in 10 mM PBS (pH 7.4) at 60 °C for 72 h in the presence of lactose (W-LT-La) and in the absence of lactose (W-LT). High temperature dry heating of BLG was conducted at 130 °C for 10 min in the presence of lactose (D-HT-La) and in the absence of lactose (D-HT). Prior to heat treatment, the BLG solution was lyophilised and a_w_-level was adjusted to 0.53 over saturated sodium bromide solution. For low-temperature dry heating, BLG was heated at 50 °C for 9 h at aw 0.65 in the absence of lactose (D-LT) and the presence of lactose (D-LT-La). Humidity was monitored with a humidity control chamber (HCP108, Memmert, Schwabach, Germany). After heat processing, dry-heated samples were dissolved in water to starting protein concentration. All samples were centrifuged at 2900× *g* for 30 min to remove insoluble material and unreacted lactose was removed by dialysis. Protein concentration of samples showing the formation of insoluble material was determined with DUMAS as described by Deng et al. [1].

### 2.4. Quantification of CML Using uHPLC-MS/MS

CML was quantified using uHPLC-ESI-MS/MS according to a method described by Troise et al. [18]. Samples were diluted to 2.5 mg/mL in ultrapure water and mixed with hydrochloric acid to a final ratio of 0.63 mg protein/1 mL 6 M hydrochloric acid. Solutions were saturated with nitrogen and heated for 22 h at 110 °C. Hydrolysates were centrifuged (4500× *g*, 10 min, 20 °C) using a Heraeus multifuge X3R (Thermo Fisher Scientific, Waltham, MA, USA) and filtered through a 0.2 µm Polytetrafluoroethylene (PTFE) syringe filter (Phenomenex, Torrance, CA, USA). An aliquot was dried under nitrogen and dissolved to the same volume in ultrapure water. Samples were centrifuge (10,000× *g*, 20 min, 20 °C) using an Eppendorf multifuge 5430R (Eppendorf, Hamburg, Germany). Subsequently, they were diluted with acetonitrile to reach 50% acetonitrile and spiked with internal standard CML-d2.

Standard solutions were prepared in a concentration range between 25 ng/mL and 750 ng/mL and spiked with CML-d2. Final concentration of CML-d2 in all sample and standard solutions was 250 ng/mL. CML was separated on a Kinetex 2.6 µ HILCI 100A, 100 × 2.1 mm (Phenomenex, Torrance, CA, USA) at 35 °C column temperature. Eluent A was ultrapure water with 0.1% formic acid, eluent B was acetonitrile with 0.1%, and eluent C was 50 mM ammonium formate. Flow rate was set to 0.4 mL/min using the following gradient (time (min)/eluent B (%)/eluent C (%)): (0/80/10), (0.8/80/10), (3.5/40/10), (6.5/80/10), (8.0/80/10), (11/80/10). Electron ionization was conducted in positive mode. Spray voltage was set to 3500 °C, vaporizing temperature was 250 °C, and sheath gas pressure was 60 psig. Capillary temperature was set to 290 °C. Parent mass [M+H]^+^ 205.3 m/z were selected in Q1 and the characteristic product ions 130.0 m/z (CE: 12 V; tube lens: 78) and 84.0 m/z (CE: 22 V; tube lens: 78) were recorded in Q3.

### 2.5. SDS-PAGE Gelelectorphoresis

Gel electrophoresis under non-reducing conditions was performed to monitor protein aggregates formed during heat treatment of BLG. Samples, NuPAGE^®^ LDS sample buffer (4× conc.) and ultrapure water were mixed in a ratio 5/5/10 (*v*/*v*/*v*), centrifuged (1 min, 500× *g*, 20 °C) on a Eppendorf multifuge 5430R (Eppendorf, Hamburg, Germany) and incubated for 10 min at 70 °C. BlueRay prestained protein marker was used as MW marker. Six microgram protein of each sample were loaded on NuPAGE™ 12% Bis-Tris protein gel, 1.0 mm. Gels were run at 120 V for 1.5 h using NuPAGE™ 1× MOPS SDS running buffer and stained with Coomassie Brilliant Blue R-250 or using Pro-Q^®^ Emerald 300 Glycoprotein staining kit according to the manufacturer’s instructions. Images of the stained gels were obtained using a Universal Hood III (Bio-Rad, Hercules, CA, USA) and Image Lab 4.1 software (Bio-Rad, Hercules, CA, USA).

### 2.6. Thioflavin-T Assay

Thioflavin-T (ThT) assay was conducted to monitor the formation of fibril structures during heating. Protein concentration was adjusted to 0.25 mg/mL and mixed with 3.9 mM aqueous ThT solution in a ratio 5.8/1.0 (*v*/*v*). All samples were prepared in duplicate. The solution was transferred in a 96-well black greiner polystyrene plate (Greiner CELLSTAR^®^, Kremsmünster, Austria). After incubated for 10 min in the dark, fluorescence emission was measured (λ_exitation_ = 450 nm, λ_emission_ = 485 nm, gain 100) using Infinite^®^ 200 PRO NanoQuant with i-control software (Tecan, Männedorf, Switzerland). The fluorescence intensity [a.U.] of heated and glycated BLG was corrected for the blank (PBS at pH 7.5).

### 2.7. Inhibition sRAGE ELISA

Inhibition sRAGE ELISA was conducted to determine sRAGE binding affinity as described by Liu et al. [13] with some modifications. Briefly, soy protein extract glycated with glucose (90 min, 100 °C, wet conditions) was used as coating material. Transparent high binding ELISA plate (Greiner Bio-One, Kremsmuenster, Austria) were coated with G90 for 12 h at 4 °C. Sample protein concentration was adjusted to 25 µg/mL with 1.5% BSA (*v*/*w*) in 0.025% tween in 10 mM PBS (PBST). The optimal protein concentration was chosen based on a dilution curve of BLG-NT obtained from a pre-experiment. The samples were pre-incubated with 1 µg/mL sRAGE in a ratio 1:1 (*v*/*v*) for 45 min at 37 °C on a NuncTM polystyrene plate (Thermo Fisher Scientific, MA, USA) before addition to the ELISA plate. The coated ELISA plate was blocked with PBS with 3% BSA (*v*/*w*) for 1 h at room temperature and washed with PBST. The washing step was repeated after each step of ELISA. After blocking, the pre-incubated sRAGE/sample mixture was transferred into the ELISA plate and incubation was continued for 1 h at 37 °C. After washing, anti-sRAGE antibody was added at a concentration of 0.25 µg/mL and the plate was incubated under shaking for 30 min at room temperature. After washing, anti-mouse polyclonal goat HRP conjugated antibody at a concentration 0.25 µg/mL was added and the incubation was continued for 30 min at room temperature. The signal was detected with TMB. The color reaction was measured at 450 nm vs. 620–650 nm reference using a Filter Max F5 multi-mode microplate reader (Molecular Devices, San Jose, CA, USA). Each sample was measured in triplicate. Amyloid-β was used as a positive control, while ovalbumin was used as a negative control.

Inhibition was calculated using the following formula:

Inhibition [%] = (Abs_max_ − (Abs_sample_ − Abs_Min_))/Abs_Max_ × 100

where Abs_Max_ is the absorbance obtained from sRAGE without competition agent and Abs_Min_ is the absorbance obtained from blank sample (PBS) without sRAGE, Abs_sample_ is the absorbance obtained from the mixture of sRAGE and each sample. High inhibition indicates high sRAGE binding affinity.

### 2.8. sRAGE Western Blot

SDS-PAGE was conducted as described before. However, protein concentration increased to 20 µg protein for wet-heated BLG at high temperature, 25 µg protein for wet-heated BLG at low temperature, 10 µg protein for dry-heated BLG in the presence of lactose, and 4 µg protein for BLG-NT and BLG heated in a dry system in the absence of lactose, to achieve similar band densities on the gel. Gels were blotted on Amersham^TM^ Protran^TM^ 0.45 µm nitrocellulose membrane (GE Healthcare Life science, Marlborough, MA, USA). Gels were blotted with semi-dry western blot blotting buffer at 15 V for 35 min. Membranes were washed for 2 × 5 min with 1x Tris buffered saline (TBS) with 0.2% tween (TBST) and incubated for 1 h at room temperature with 3% BSA in TBST. Subsequently, membranes were washed 2 × 10 min with TBST and incubated at 4 °C for 12 h with sRAGE diluted to 1 µg/mL with 1.5% BSA in TBS (*w*/*v*). Membranes were washed 4 × 7 min with 1 × TBST/Triton and 2 × 5 min with TBST. Anti-RAGE antibody was diluted to 0.25 µg/mL with 1.5% BSA in TBS and added to the membrane for 1 h at room temperature. Subsequently, the membrane was washed as described before and incubated for 1 h with anti-mouse polyclonal goat HRP conjugated antibody diluted to 0.25 µg/mL with 1.5% BSA in TBST. After incubation, membranes were washed 4 × 7 min with 1 × TBST/Triton and 2 × 5 min with TBS. ECL western blot detection reagent was added for 30 s. Chemiluminescence was visualized in ChemHighsensitivity mode using an Universal Hood III (Bio-Rad, Hercules, CA, USA) and Image Lab 4.1 software (Bio-Rad, Hercules, CA, USA)

### 2.9. CML Western Blot

SDS-PAGE western blotting was performed as described before. Blotted membranes were washed 2 × 5 min with TBST and incubated 1 h in TBST with 3% BSA. After washing 2 × 10 min with TBST, membranes were incubated with 0.25 µg/mL anti-CML-antibody with 1% BSA and 0.5% raw whey protein in TBS at 4 °C for 12 h. Membranes were washed 4 × 7 min with TBST/Triton and 2 × 5 min with TBST. Anti-mouse polyclonal goat HRP conjugated antibody was diluted to 0.25 µg/mL with 1% BSA and 1% raw whey protein in TBST. The membrane was incubated for 30 min with the antibody. Subsequently, it was washed 4 × 7 min with TBST/Triton and 2 × 5 min with TBS. ECL chemiluminescence detection was conducted as described before.

### 2.10. sIgE Binding Dot Blot

sIgE Dot blot was conducted to screen the available sera and plasma for their binding affinity to BLG heated under dry conditions at high temperature and BLG heated under wet conditions at high temperature, each time in the presence of lactose or absence of lactose.

Each time, 5 µg protein was spotted on the membranes. The membranes were washed 5 min with TBST and incubated for with 3% BSA in TBST for 1 h at room temperature. Membranes were washed 2 × 5 min in TBST and incubated with sera/plasma diluted in 1% BSA/TBST for 12 h at room temperature. Sera dilutions were prepared in the ratios as shown in Table 1. Membranes were washed 4 × 7 min with TBST/Triton and 2 × 5 min with TBST. Mouse anti-human sIgE antibody was diluted 0.5 µg/mL with 0.5% non-fat dry milk (NFDM) in TBST and added to the membranes for 1 h at room temperature. Subsequently, the membranes were washed 4 × 7 min with TBST/Triton and 2 × 5 min with TBST. Anti-mouse polyclonal goat HRP conjugated antibody was diluted to 0.25 µg/mL with 0.5% NFDM in TBST and added to the membranes for 30 min at room temperature. Subsequently, they were washed 4 × 7 min with TBST/Triton and 2 × 5 min with TBS. Chemiluminescence detection was conducted as described before.

### 2.11. sIgE Binding Western Blot

sIgE western blot was conducted to directly identify the bands on the SDS-PAGE that show sIgE binding. The specimen was selected depending their activity observed in the sIgE Dot blot and pooled for similar sIgE binding according to the scheme in Table 2.

SDS-PAGE was prepared and blotted as described for sRAGE western blot. All other steps were conducted as described for sIgE Dot blot.

### 2.12. Statistical Analysis

Statistical analysis was conducted using SPSS version 23. For multiple sample comparison, one-way analysis of variance (ANOVA) and Tukey post hoc comparison test was used. Results were considered statistical different at *p* < 0.05 if not mentioned otherwise.

## 3. Results

To investigate the effect of different heat treatments of BLG on aggregation, formation of MR products, binding to sRAGE, and binding of sIgE, BLG was heated in solution (wet conditions) and in powdered form with controlled water activity (dry conditions). For each humidity condition, BLG was heated in the presence or absence of the milk sugar lactose to distinguish between the effect of glycation and heating. Additionally, each treatment was conducted below the denaturation temperature (low-temperature heating) and above the denaturation temperature (high temperature heating) of BLG.

### 3.1. Solubility of BLG after Heat Treatments

Solubility of BLG was impaired after heating in a dry system, but only in the presence of lactose at 130 °C. This was observed by the formation of insoluble material after dissolving BLG in PBS. Therefore, the insoluble aggregates were removed by centrifugation and the soluble fraction of was used for further characterization. Analysis of the total nitrogen content with DUMAS showed a recovery of soluble protein of 76.5% of the total protein. BLG heated in a wet system and at low temperature in a dry system, did not show the formation of insoluble material, independent of the presence of lactose.

### 3.2. Quantification of Nε-carboxymethyl-L-lysine

CML was quantified in all samples to determine the level of the advanced stage of the MR. Table 3 compares CML quantities of BLG heated under different conditions in the presence or absence of lactose. No CML was found in the BLG samples that were heated without lactose, because CML formation requires the presence of either a reducing sugar moiety or polyunsaturated fatty acids. CML quantities were on average ~30% lower when BLG was heated in a wet system than when it was heated in a dry system. The quantities of CML were positively correlated with the temperature of heating in both systems. Additionally, CML levels were relatively higher after dry heating of BLG compared to wet heating.

The levels of CML, as a commonly used marker for the “advanced stage” of the MR, indicate that the level of glycation was higher when BLG was heated under dry conditions than under wet conditions. The formation of CML was further facilitated at higher temperature heating within the same humidity conditions.

### 3.3. SDS-PAGE

**Figure 1:**
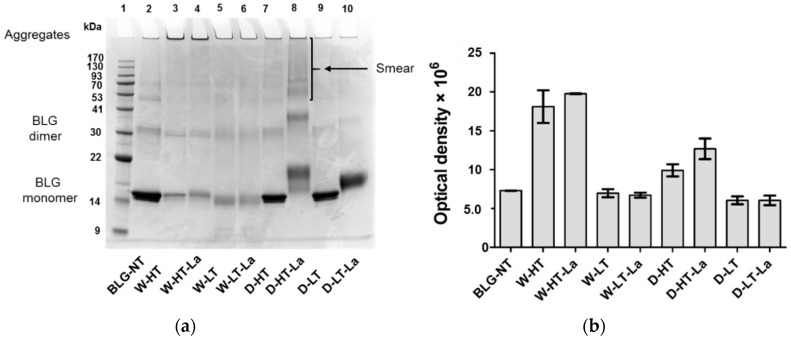
(**a**) Non-reducing SDS-PAGE image of soluble BLG heated and glycated in a wet system (W) and in a dry system (D), respectively in the absence of lactose or in the presence of lactose (La) and at either high temperatures (HT) or low temperatures (LT). (**b**) optical density of the aggregates visible in the top of each lane (>170 kDa). Error bars represent standard deviation of the optical density of two SDS-PAGE gels prepared independently from each other.

Protein aggregation was monitored using SDS-PAGE under non-reducing conditions, as shown in Figure 1.

When heated in a wet system, BLG formed high-MW aggregates that did not migrate into the gel. Aggregate formation occurred below (60 °C, Lanes 5–6) as well as above the denaturation temperature of BLG (100 °C, Lanes 3–4); however, they were more abundant when BLG was heated above its denaturation temperature. The relative aggregate quantities, based on the optical density, showed this effect of heating temperature, but not of lactose presence for the wet-heated samples (Lanes 3–4 and 5–6 of Figure 1a, corresponding bars of Figure 1b). Dry-heated BLG aggregates showed 23% higher optical density in the presence of lactose compared to the heating in the absence of lactose, however only when BLG was heated at high temperature.

Heating of BLG in a dry system resulted in protein aggregation showing clear differences between the samples with and without lactose. After heating of BLG with lactose at high temperature (Figure 1a, Lane 8), the MW of the BLG monomer increased by approx. 4 kDa while the BLG dimer showed an increasing MW by approx. 6 kDa. The relative intensity of the BLG dimer increased by 55% compared to the unheated control, indicating a shift of the monomer-dimer equilibrium of BLG. Additionally, D-HT-La showed a smear starting from an apparent MW > 53 kDa until the top of the gel (Lane 8). D-LT (Lane 9) showed monomeric BLG at an apparent MW of 14 kDa, which is similar to the non-treated BLG (BLG-NT). However, a faint smear, similar to the one of D-HT-La but with much lower intensity, was also observed for this sample. Dry heating at low temperatures did not induce the formation of aggregates, neither in the presence or absence of lactose, in the soluble fraction. However, a shift of apparent MW of the BLG monomer by 2 kDa was observed in D-LT-La.

These data indicate that whereas wet heating favors formation of aggregates, dry heating favors MR.

### 3.4. Detection of Glycation Strucutres on SDS-PAGE Visible Proteins

**Figure 2:**
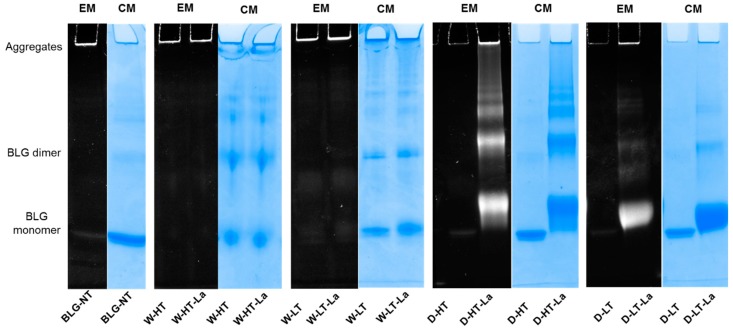
Non-reducing SDS-PAGE image of soluble BLG heated and glycated in a wet system (W) and in a dry system (D), respectively in the absence of lactose or in the presence of lactose (La) and at either high temperatures (HT) or low temperatures (LT). Gels were stained with Q^TM^ Emerald 300 staining for glycoproteins (EM) and Coomassie staining (CM) for total protein staining.

To directly identify which of the bands visible on the SDS-PAGE are modified by MR, the Pro-Q™ Emerald 300 glycoprotein staining kit was used. The staining reacts with structures carrying a reducing carbonyl moiety and can therefore indicate the presence of Maillard reaction products (MRPs) from all three stages of the MR. Stained glycated protein will appear as white bands on the SDS-PAGE image (Figure 2).

**Figure 3:**
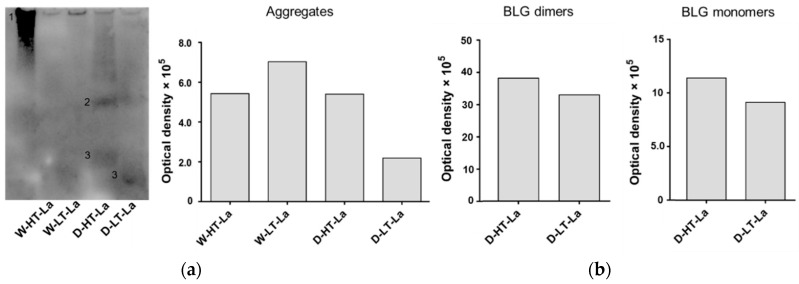
(**a**) Membrane image of CML western blot measured of BLG heated in the presence of lactose (La) in a wet system (W) or a dry system (D) at low temperatures (LT) or high temperatures (HT). G90: soy protein extract glycated with glucose for 90 min (positive control). Numbers indicate aggregates (1), BLG dimers (2), and BLG monomers (3). (**b**) Optical density of bands visible on the CML western blot categorized in high-MW aggregates, BLG dimers, and BLG monomers as indicated on the membrane.

Wet-heated BLG showed a fluorescence signal of the aggregates in the top of the gel, independent of the presence of lactose and the temperature. Based on CML western blot (Figure 3) it can be concluded that under wet heating conditions, the glycation structures are mainly present in the high-MW aggregates; however it was not clearly visualized by glycation staining. It may be explained by the fluorescent specking at the edges of the gel which is an intrinsic property of the dye and/or the fluorescence signal from tryptophan residues of native BLG. Both effects can contribute to the positive signal for unheated BLG in the top of the gel. It may also partly contribute to the positive signal of the high-MW aggregates of wet-heated BLG making difficult to quantitatively interpret the presence of reducing carbonyl groups in the wet-heated samples.

Dry heating showed high fluorescence when heated in the presence of lactose at high and low temperatures, and not when heated in the absence of lactose. The signal for glycation structures was observed for all proteins that were also visible with Coomassie staining. A positive signal with glycation staining was also observed for the BLG monomer of D-HT and D-HT-La and the high-MW aggregates. The same is also true for BLG-NT. However, the relative glycation staining intensity of the aggregates from heated and glycated BLG is higher than the high-MW band visible in BLG-NT.

The band of wet-heated BLG at low temperature showed higher intensity than wet-heated BLG at high temperature, which is in contradiction to the total CML quantities. This can be explained by the lower accessibility of the CML antibody to CML structures if they are buried inside of the aggregate. This effect might have more impact in high temperature treated samples because of the more compact structure of the aggregates. These data show that under wet heating conditions glycation structures and CML are mainly detected in high-MW aggregates, while under dry heating conditions they are observed in all protein factions.

### 3.5. Formation of Fibril Structures

**Figure 4:**
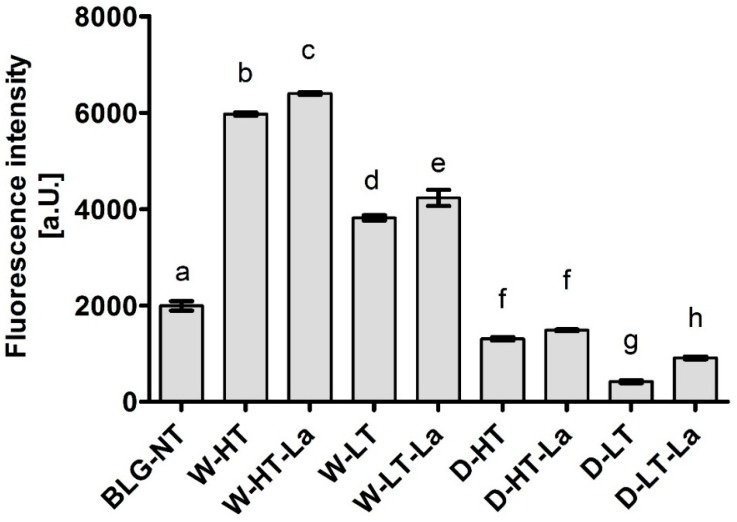
ThT fluorescence intensity after incubation with BLG heated in the absence of lactose or in the presence of lactose (La) in a wet system (W) or a dry system (D) under low temperatures (LT) or high temperatures (HT). BLG-NT: unheated BLG. Error bars represent standard deviation of technical duplicates. Letters indicate significant differences between the BLG groups. *p*-values < 0.05 are considered statistically significant, as analyzed with one-way ANOVA and Tukey post hoc comparison test (SPSS).

The ThT-assay was conducted to monitor the formation of fibril structures and exposure of β-sheet structures upon heating and glycation of BLG. Results are shown in Figure 4.

Highest fluorescence intensity was observed for wet-heated BLG and increased with higher heating temperature. When heated under dry conditions, the fluorescence intensity did not increase above the level of BLG-NT, indicating that no additional β-sheet structures were exposed. These results indicate that β-sheet structures are exposed and/or formed when BLG is heated under wet conditions independent of the presence of lactose. At the same time, this effect is not observed under dry heating conditions. This indicates that the exposure of β-sheet structures is facilitated under wet heating conditions while the presence of lactose plays a minor role independent of the humidity conditions.

### 3.6. Binding of sRAGE to Heated and Glycated BLG

**Figure 5:**
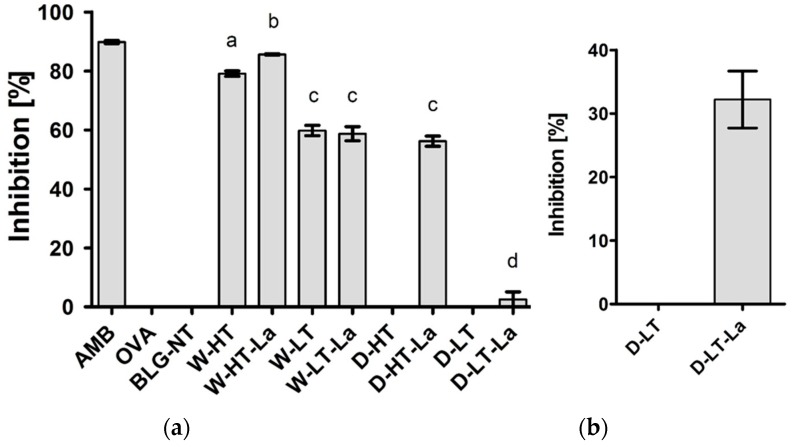
(**a**) Inhibition of sRAGE binding in competition ELISA by BLG heated in wet system (W) and dry system (D) in the absence of lactose or presence of lactose (La) at high or low temperature (HT and LT) for all treatments at a protein concentration 25 μg/mL. AMB: amyloid-β (positive control), OVA: ovalbumin (negative control). (**b**) Results of inhibition sRAGE ELISA for D-BLG-LT and D-LT-LA at a protein concentration 100 μg/mL. Error bars represent standard deviation of technical triplicates. Letters indicate significant differences between the BLG groups. *p*-values < 0.05 are considered statistically significant, as analyzed with one-way ANOVA with Tukey post hoc comparison test (SPSS).

Binding of sRAGE to heated and glycated BLG was monitored using sRAGE inhibition ELISA where high inhibition indicates high sRAGE binding affinity (Figure 5).

**Figure 6:**
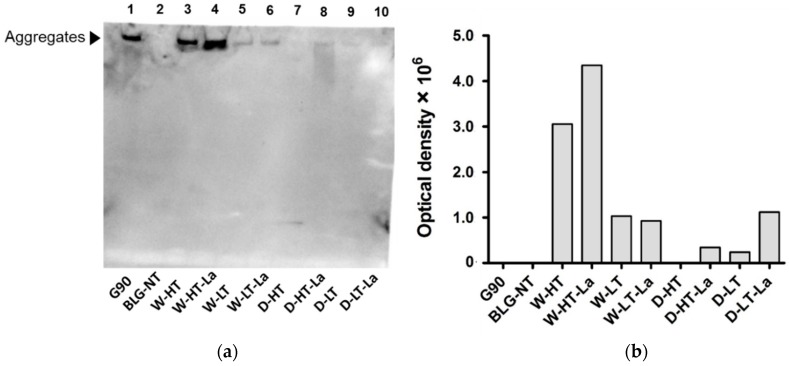
(**a**) Membrane of sRAGE western blot for BLG heated in the absence of lactose or presence of lactose (La) under wet conditions (W) or dry conditions (D) at high temperatures (HT) or low temperatures (LT). (**b**) Optical density of the bands showing sRAGE binding affinity. G90: soy protein extract glycated with glucose for 90 min, 100 °C, wet conditions (positive control).

BLG-NT did not show any binding affinity to sRAGE while BLG heated in wet systems, both with and without lactose, showed increased sRAGE binding affinity compared to BLG-NT. Wet heating at higher temperature resulted in 28% higher sRAGE binding affinity than wet heating at lower temperature. At high temperature, sRAGE showed 8% higher binding to W-HT-La than W-HT, while at low temperatures no lactose dependent difference was observed. In contrast to the wet system, BLG heated without lactose in a dry system (at either high or low temperature) did not show any binding affinity for sRAGE. D-HT showed a similar sRAGE binding affinity as the low-temperature wet-heated samples. BLG heated in dry system with lactose at low temperature (D-LT-La) showed the lowest sRAGE binding compared to the other samples; however the binding increased with higher protein concentration (Figure 6b), indicating that sRAGE does show binding affinity but lower than for the other samples.

Western blot analysis was used to directly identify which fractions on the SDS-gel are responsible for the sRAGE binding determined for heated and glycated BLG in the sRAGE inhibition ELISA. These results are shown in Figure 6.

No binding of sRAGE to the monomeric form of BLG was observed in any of the heat-treated BLG samples except for dry heating at low temperature (Figure 6a, Lane 9). The faint band in the lower part of Lane 7 is most likely an artefact, as no protein was seen at this position on the SDS-PAGE. The fraction showing highest affinity to sRAGE were high-MW aggregates of BLG observed in wet-heated samples, especially those heated at high temperature (Lanes 3–4). Aggregates of wet-heated samples with lactose showed ~30% higher intensity than samples heated without lactose, suggesting that both aggregation but also glycation play a role in sRAGE binding. The same tendency of slightly increasing sRAGE binding in the presence of lactose under wet conditions, was also observed in the ELISA. At low-temperature wet heating, no increased signal intensity was observed when lactose was present. In the dry system at high temperature, sRAGE binding to the smear formed at MW > 53 kDa was observed for lactose-containing sample. At low-temperature dry heating a faint band was observed for aggregates of lactose-free heated BLG. The signal intensity of BLG heated in the presence of lactose under dry conditions, based on the optical density (Figure 6b), increased by ~75%. The relative binding intensity of BLG heated under different conditions are in line with the ELISA results (Figure 5). These results indicate that aggregation promotes sRAGE binding affinity to a greater extent than glycation.

### 3.7. sIgE Binding to Heated and Glycated BLG

**Figure 7:**
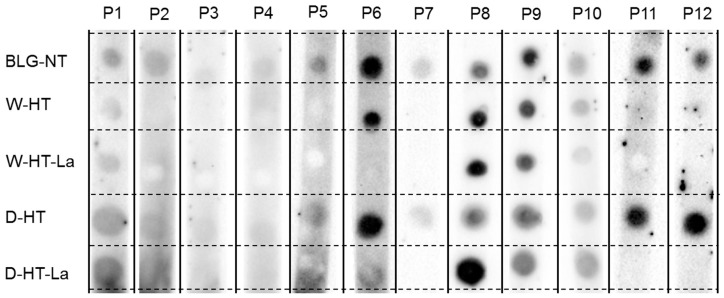
sIgE dot blot membranes of sera from 12 different patients (P) tested for their binding affinity to unheated BLG (BLG-NT) and BLG heated at high temperatures (HT) either under wet (W) or dry (D) conditions, both in the absence of lactose (BLG) or in the presence of lactose (Lac).

sIgE binding dot blot was conducted to screen the available plasma and sera for their sIgE binding affinity to untreated BLG as well as heated and glycated BLG under wet or dry conditions at high temperatures. The results of the 12 different sera/plasma samples from Table 1 are shown in Figure 7. Based on the dot blot results, the sera were pooled (see Table 2) to perform sIgE western blots.

No binding to BLG was observed for the sera of Patient 3 and only weak binding to unheated BLG for the sera of Patient 4. All other sera showed a positive binding to BLG-NT.

**Figure 8:**
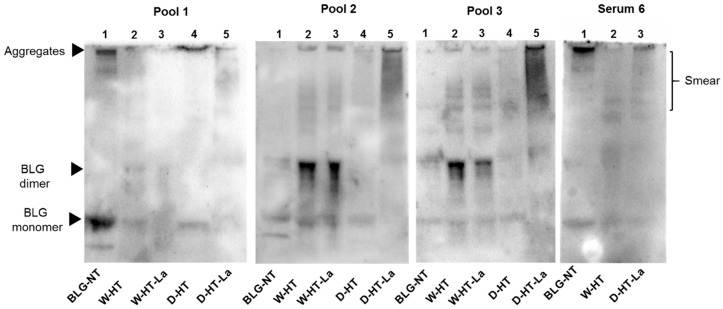
sIgE western blot membranes of unheated BLG (BLG-NT) as well as BLG heated at high temperatures (HT) either under wet (W) or dry (D) conditions, both in the absence of lactose or in the presence of lactose (La). Sera of cow’s milk allergic patients were pooled. Pool 1: Sera 7, 11, 12. Pool 2: Serum 8 and Serum 9. Pool 3: Serum 1 and Serum 10.

Four different profiles for sIgE binding to heated and glycated BLG were observed. The sera described as sIgE binding profiles 1-4 were pooled (see Table 2 for details) to perform western blots (Figure 8). Sera 7, 11, and 12 showed only binding to unheated BLG and BLG heated in the absence of lactose under dry conditions. Serum 2 and Serum 5 also showed a similar profile of binding to BLG to Sera 7, 11, and 12; however, because of the high background, it could not be excluded that they also show binding to BLG heated in the presence of lactose under dry conditions. Therefore, they were not included in Pool 1.

Western blots confirmed the results observed in dot blot and indicated that the binding of sIgE occurs to both BLG monomer and the aggregates, with strongest binding for BLG-NT and D-HT (Pool 1, Line 4 and 5). A band below the BLG monomer band was also observed in unheated BLG although this band was not seen on the SDS-PAGE (Figure 1a). While the binding intensity for BLG-NT was equal between the monomer and the aggregates, in D-HT the binding intensity to the aggregates was ~30% higher than to the monomer (Pool 1, Lane 4). Weak binding was also observed for the BLG monomer in samples heated in the absence of lactose under wet conditions and to the smear of BLG heated in the presence of lactose under dry conditions (Pool 1, Lane 2 and 5). Pool 2 showed binding to all samples, with higher binding to wet-heated BLG independent of the presence of lactose and to dry-heated BLG in the presence of lactose. Western blots showed sIgE binding to the BLG monomer and the BLG dimer in unheated BLG. When heated under wet conditions (Pool 2, Lane 2 and Lane 3), binding was observed to the BLG monomer, BLG dimer, and the aggregates. The dimer showed the highest sIgE binding intensity with ~50% and ~60% of the total optical density of W-HT and W-HT-La, respectively. BLG heated under dry conditions showed binding to the BLG monomer and to a smear > 53 kDa, with higher binding when heated in the presence of lactose (Pool 2, Lane 5) compared to heating in the absence of lactose (Pool 2, Lane 4). Additionally, in the presence of lactose, the aggregates also showed high sIgE binding. Pool 3 showed equal binding to unheated and all heated BLG. This sample showed the same sIgE binding pattern as Pool 2, except for the smear of D-HT-La (Pool 3, Lane 5) that showed higher intensity than in Pool 2. Serum 6 showed a unique binding pattern with strong binding to unheated BLG, and BLG heated in the absence of lactose for dry and wet heating conditions. A weak binding was also observed for BLG heated under dry conditions in the presence of lactose but not to the equivalent sample heated under wet conditions. Interestingly, the binding intensity was higher to the aggregates than to the monomer, for both BLG-NT (Serum 6, Lane 1) and D-HT (Serum 6, Lane 3) with ~30% and ~55%, respectively. For BLG heated under wet conditions in the absence of lactose, the binding was also observed to the monomer and dimer but less to the aggregates.

To summarize, the results of sIgE dot blot indicate high individual variations in the sIgE binding profiles to heated and glycated BLG and allowed the grouping into four binding affinity profiles. Wet-heated BLG showed reduced sIgE binding when compared to non-treated BLG for 5 out of 10 sera giving the positive signal, independent of the presence of lactose. Dry heating of BLG without lactose did not affect sIgE binding compared to non-treated BLG. On the other hand, BLG dry heated in the presence of lactose increased sIgE binding compared to both untreated BLG and dry-heated BLG without lactose, revealing an effect of glycation on the sIgE binding capacity. Results showed which BLG fractions were responsible for sIgE binding observed in the dot blot analysis, indicating a shift of sIgE binding towards higher MW fractions (BLG dimers and aggregates) formed upon heating.

## 4. Discussion

Food processing promotes the changes of the immunogenic and antigenic properties of food allergens [3], among others via MR. In this study, we showed that during processing of BLG both the temperature and water activity (wet vs. dry heating) are important factors affecting its immunogenicity. Wet heating of BLG, independent of the presence of lactose in the sample, promotes the formation of aggregates recognized by sRAGE and sIgE from CMA patients. In contrast to wet heating the aggregates formed upon dry heating showed differences in an immunogenic profiles of the samples heated in the presence or absence of lactose, therefore revealing contribution of glycation to the formation of both sRAGE and sIgE binding epitopes.

Both glycation and aggregation of BLG depends on the applied heating conditions. Glycation of milk proteins is promoted at low water activity of 0.31–0.98 [19]. At the same time, heating at high humidity conditions facilitates the aggregation of BLG via disulfide linkages [20,21] but leads to a relative lower levels of glycation structures compared to dry heating [22,23]. This effect was also observed in this study where the formation of CML and detection of glycation structures on BLG was significantly higher in dry-heated BLG than in wet-heated BLG. This was confirmed by CML western blot (Figure 3) showing highest levels of CML in D-HT-La detected in monomers, dimers, and aggregates of BLG while in the wet-heated samples the CML appeared only in the aggregates. Next to that, more glycation structures formed during heating of BLG under dry conditions in the presence of lactose were detected by the glycation staining (Figure 2). On the other hand, the formation of high-MW aggregates was independent of the presence of lactose in wet-heated samples compared to dry-heated samples. However, aggregation was also observed in the sample heated under dry conditions with lactose. It has already been described that glycation-induced modifications of lysine and arginine can increase the flexibility of the protein structure and leads to the formation of covalent cross linking between different amino acid residues. Both facilitates protein denaturation and aggregation [16,24] and influences the morphology of the protein aggregates [25,26]. Additionally, glycation strongly affects the size of these aggregates when heated under dry conditions but may also contributes to structural differences of the formed aggregates under both humidity conditions [1]. However, reactant mobility during the wet heating also affects the aggregation rate of proteins, where higher inter-molecular mobility results in higher protein aggregation [27]. The observed differences in aggregation of wet-heated BLG vs. dry-heated BLG are in line with the findings of Deng et al. [1] who showed increased formation of polymers and oligomers in wet-heated BLG compared to dry-heated BLG. The authors also showed that the dry heating of BLG at 130 °C increased its oligomerization and polymerization and was mainly affected by the presence of lactose. On the other hand, the wet heating led to more exposure of β-sheet structures in the soluble aggregates than dry heating. These findings stay in line with our results showing a higher content of surface-exposed β-sheets structures in the wet-heated BLG independent of the presence of lactose in the sample. The formation of aggregates in both wet and dry heating conditions was shown to have a great impact of the formation of sRAGE binding ligands. Three different patterns of sRAGE binding affinity (Figure 6) were observed, depending on the heating temperature and the water activity of the system. First, when heated in the wet system high sRAGE binding was observed to the formed aggregates, regardless of the presence of lactose. Secondly, sRAGE binding occurred independent of the presence of CML but increased with higher exposure of β-sheet structures after wet heating (Figure 3 and Figure 4). Finally, in the dry-heated system, sRAGE binding was observed only to BLG heated in the presence of lactose (high-MW fractions), revealing an impact of glycation under dry heating conditions (Figure 6). Being a promiscuous receptor, sRAGE binds not only to AGEs but also to amyloid-β, S100 protein, and HGMB1 [11]. Those ligands share a common property to act as oligomers, highlighting the potential of sRAGE to recognize high-MW structures [12]. Next to the aggregation itself, the glycation-induced aggregation was already pointed out to be responsible for the formation of sRAGE ligands under dry heating conditions; however this was not directly proven [13]. With the western blot analysis of sRAGE binding we confirmed these findings, proving that under the dry heating conditions of BLG glycation without aggregation does not lead to increased sRAGE binding. Consequently, the changes in sRAGE binding were accompanied by the changes in sIgE binding affinity by (treatment-dependent) formation of neo-epitopes or destruction of existing epitopes. Several studies indicated that both glycation and aggregation, contribute to changes in cellular recognition and allergic sensitization [1,4,13,28,29]. The results of dot blot with the sera of CMA patients showed high individual variations in the sIgE binding to heated and glycated BLG. Four different profiles of sIgE binding affinity were observed which showed, depending on the used conditions, either increased or diminished sIgE binding to heat-treated BLG. The dot blot analysis revealed that wet heating reduces sIgE binding independent of the presence of lactose which was observed in 5 out of 10 sera giving a positive signal with non-treated BLG (Figure 7). Therefore, glycation appears to play a minor role in changing sIgE binding when BLG is heated under wet conditions. For dry-heated samples, sIgE binding was only decreased when lactose was present during heating (Figure 7), indicating the masking of epitopes by the attached sugar moiety. These differences indicate that humidity applied during the heat processing as well as glycation are important factors influencing sIgE binding affinity. The western blot results (Figure 8) obtained for four pools of sera to heated BLG allowed to detect the fractions and bands responsible for sIgE reactivity observed in dot blot. Three different fractions present in non-treated BLG were recognized by sIgE:BLG monomer, BLG dimer, and oligomers in the top of the gel. Binding of sIgE to the oligomers present in non-treated BLG was always accompanied with the positive binding to the oligomers present in dry-heated sample without lactose suggesting the existence of similar epitopes in these two samples (Figure 8, Pool 1 and Serum 6). Interestingly, the aggregates formed during the wet heating showed sIgE reactivity only in case of half of the tested sera indicating a lactose-independent decrease of sIgE binding affinity. It has been shown in the past that the free cysteine (C121) is strongly involved in the aggregation of BLG when heated in a wet system and this results in the formation of covalently linked aggregates [21]. Due to the higher translational motion in the wet system compared to the dry system the formation of these covalent linkages is favored in the wet system leading to higher extend of aggregation. This might also result in the embedding or exposure of different sIgE epitopes when comparing the two humidity conditions. The observation of sIgE binding for half of the patients stay in line with a previous finding that reported decreased sIgE binding to BLG when heated above 90 °C under wet conditions [9,30,31]. Additionally, Kleber and Hinrichs [31] postulated that protein aggregation may decreases sIgE binding affinity. However, our study points out that there are different MW fractions formed during wet and dry heating of BLG that can be recognized by sIgE of some patients. This was lactose-independent after wet heating of BLG, and was mostly found to higher MW fractions > BLG dimers. The same patients also show higher sIgE binding to dry-heated BLG in the presence of lactose compared to the lactose-free control. The most immuno-reactive fraction was a high-MW smear of dry-heated BLG in the presence of lactose which also showed the presence of glycation structures and CML (Figure 8). This was also true for the BLG monomer and dimer in this sample but sIgE binding was not observed to these fractions in the same pool of sera. This indicates that MR may has a double effect in sensitization. First, it can lead to masking of epitopes in the BLG monomer and dimer as described previously also for other food allergens [8,32,33]. Secondly, the formation of glycation-induced aggregates can enhance sIgE binding via neo-epitope formation. The study of Vissers et al. [34] showed reduced allergenicity of MR-modified peanut allergen Ara h 1 in sIgE binding test while enhanced β-hexominidase release from basophils upon incubation with the same MR-modified allergen. The authors suggested that MR-induced aggregates of Ara h 1 could be responsible for the observed increased capacity of antigen to cross-link the sIgE and initiate the mediator release from RBL-2H3 cells. Similar observations were also made for other protein families such as peanut proteins and scallop tropomyosin [8,35,36,37]. Thus, the influence of MR on sIgE binding seems to depend on physicochemical properties of proteins (hydrophobicity, size, amino acid composition, charge) as well as on the conditions of the MR (type of sugar, time, water activity, pH, temperature, presence of salts) [36,38].

Different sIgE binding profiles observed in this study indicate the high individual differences between CMA patients and demonstrates the importance of involving better characterized CMA patients’ sera in the sIgE binding study. That would allow a prediction to be made towards the influence of aggregation and glycation of BLG on its allergenicity. This would help to optimize processing conditions towards less immunoreactive products which is especially relevant in the production of infant formula. Furthermore, it should be taken into account that both aggregation and glycation affect protein digestibility. Antigens with resistance to gastrointestinal digestion are commonly understood to be the most potent allergens in terms of sIgE binding [39]. It is known that glycation-induced aggregates are more resistant to in vitro gastrointestinal digestion [26]. Therefore, aggregates might have higher physiological relevance than aggregates formed in the absence of sugar even though they were as potent in stimulating sRAGE binding and sIgE binding as aggregates formed in the absence of sugar.

## 5. Conclusions

sRAGE binding is highest when BLG is heated under wet conditions and is mostly determined by aggregation rather than formation of MRPs. Under dry heating conditions sRAGE binding is mostly affected by the presence of lactose but a strict distinction between the effect of the formation of MRPs and glycation-induced aggregation could not be made. Also sIgE binding seems to be affected by aggregation and glycation; however high inter-individual differences do not allow clear differentiation between the effects of the used heating conditions on allergic sensitization. Therefore, more individuals need to be screened to get more insight in the role of aggregation and glycation on sRAGE binding and sIgE binding affinity of thermally processed BLG. However, this data suggests that different heating conditions of BLG result in the formation of different sRAGE ligands and sIgE epitopes and that the water activity during the processing of milk compounds contributes to the pattern of sensitization.

## Figures and Tables

**Table 1:** sIgE levels of sera from cow’s milk allergic patients. sIgE levels as measured by ImmunoCap.

Patient #	sIgE-Level Cow’s Milk Proteins [kU/L]	Specimen Type	Dilution
1	52.2	Serum	1:5
2	0.73	Serum	1:3
3	0.96	Serum	1:3
4	0.53	Serum	1:3
5	0.96	Serum	1:3
6	1.69	Serum	1:3
7	1.55	Serum	1:3
8	>100	Plasma	1:5
9	91.0	Plasma	1:5
10	94.8	Plasma	1:5
11	28.4	Serum	1:5
12	6.6	Serum	1:5

**Table 2:** Pooling of sera and plasma for IgE-western blot and used dilution. Membrane number indicates the membrane that was incubated with the specific pooled specimen.

Pool/Serum	Patient Serum/Plasma	Dilution
Pool 1	7, 11, and 12	1:5
Serum 6	6	1:7
Pool 2	8 and 9	1:7
Pool 3	1 and 10	1:5

**Table 3:** CML quantities determined by uHPLC-ESI-MS/MS in the soluble fraction of BLG heated in the presence of lactose (La) using wet (W) or dry (D) heat treatment at either low or high temperature (LT/HT), BLG-NT: unheated BLG, ND: quantities below the detection limit.

Sample	BLG-NT	W-HT-La	W-HT	W-LT-La	W-LT	D-HT-La	D-HT	D-LT-La	D-LT
CML mg/100 g protein	ND	129 ± 10 a	ND	77 ± 4 b	ND	182 ± 7 c	ND	158 ± 9 d	ND

Data are expressed in means of technical duplicates ± SD (*n* = 2). Letters indicate significant differences between groups (*p* < 0.05) as analyzed by one-way ANOVA and Tukey post hoc comparison test (SPSS).
